# Myeloma bone disease imaging on a 1st-generation clinical photon-counting detector CT vs. 2nd-generation dual-source dual-energy CT

**DOI:** 10.1007/s00330-022-09225-0

**Published:** 2022-11-09

**Authors:** Moritz T. Winkelmann, Florian Hagen, Lucie Le-Yannou, Jakob Weiss, Philipp Riffel, Ralf Gutjahr, Sebastian Faby, Konstantin Nikolaou, Marius Horger

**Affiliations:** 1 Department for Diagnostic and Interventional Radiology, University Hospital Tübingen, Hoppe-Seyler-Straße 3, 72076 Tübingen, Germany; 2 Department for Diagnostic and Interventional Radiology, University Hospital Freiburg, Freiburg, Germany; 3 Institute of Clinical Radiology and Nuclear Medicine, University Hospital Mannheim, Heidelberg University, Mannheim, Germany; 4 Siemens Healthcare GmbH, Forchheim, Germany

**Keywords:** Photon counting CT, Dual source CT, Multiple myeloma, Spatial resolution, Image quality

## Abstract

**Objective:**

Subjective and objective image quality comparison of bone microstructure and disease-related abnormalities in multiple myeloma patients using a 1st-generation dual-source photon-counting detector CT(DS-PCD-CT) and a 2nd-generation dual-source dual-energy (energy-integrating detector) CT (DS-EID-CT).

**Methods:**

Fifty multiple myeloma patients (mean age 67.7 ± 10.9 years,16 females) were prospectively enrolled. Unenhanced whole-body CTs were clinically indicated and performed on DS-EID-CT and DS-PCD-CT (median time difference: 12 months). DS-PCD-CT was performed in Quantumplus UHR mode and DS-EID-CT was performed using dual-energy mode. DS-PCD-CT kernel was set at Br64 with Quantum iterative reconstruction strength Q1; for DS-EID-CT a comparable I70f kernel with SAFIRE iterative reconstruction strength 1 was used. Two independent radiologists assessed image quality subjectively using a 5-point Likert scale considering delineation and sharpness of trabecular bone and lytic bone lesions in the spine and pelvic bones. Additionally, ImageJ was used for quantification of bony septa inside the cancellous bone and through or the edges of osteolysis.

**Results:**

Overall quality as well as detectability and sharpness in the delineation of lytic bone lesions were superior for DS-PCD-CT compared with DS-EID-CT (*p* < 0.0001). The inter-reader agreement for subjective image quality readings showed excellent consistency(*α* = 94.2–98.8). CTDI and DLP mean values for DS-PCD-CT and DS-EID-CT were 1107.4 ± 247.6 mGy*cm and 8.2 ± 1.8 mGy vs. 1344.3 ± 204.6 mGy*cm and 10.1 ± 1.9 mGy. The quantitative metric for bone microstructure in the femoral head showed significantly better visualization of trabeculae in DS-PCD-CT compared with DS-EID-CT (*p* < 0.0001). Quantitative analyses of edge sharpness of osteolysis showed significant steeper edges for DS-PCD-CT (*p* < 0.0001).

**Conclusion:**

DS**-**PCD-CT significantly improves spatial resolution of bony microstructure and lytic bone lesions compared to DS-EID-CT.

**Key Points:**

*• Application of photon-counting detector CT is superior to dual-source dual-energy integrating detector in clinical workup of multiple myeloma patients.*

*• Compared to energy integrating detectors, photon-counting detectors significantly increase the spatial resolution of bone microstructure including disease-related lytic bone lesions in patients with multiple myeloma.*

## Introduction

Multiple myeloma is a malignant hematologic disease of the mature B-cells primarily affecting the bone marrow. However, early in the disease course, medullary tumor cell expansion paired by complex paraneoplastic pathophysiologic mechanisms affecting bone metabolism aggresses also the bone (myeloma bone disease) leading to bone destruction in a diffuse or focal manner, mostly combined [1]. Affection of the skeleton has two major implications [2]. One is the tumor staging according to the Salmon and Durie classification from 1975 (bony lysis as a surrogate marker for marrow infiltration; staging depends on the number of lesions) which has immediate therapeutic implications whereas the other one is related to the risk of fracture and related neurologic complications [3]. For this purpose, an accurate skeletal evaluation is mandatory. Classically, this task was managed by plane radiographs, but in modern times CT has advanced to the method of first choice due to its superior spatial resolution and absence of image superimposition [4–6]. Nevertheless, even CT has its limitations which are in part methodically inherent (inferior bone marrow sensitivity, e.g., over MRI) and in part derived from protocol constrains (low dose, high pitch, low spatial resolution, etc.) [7, 8]. However, a more accurate and earlier detection of bone abnormalities would be desirable for decision making. As whole-body CT-surveillance implies a large field-of-view, in-plane resolution is thereby diminished whereas the use of comb filters to achieve high-resolution imaging implies decreased radiation dose efficiency [9].

At this point, the advent of photon-counting CT-technology might represent a promising approach to overcome these limitations. The photon-counting detector measures each individual x-ray that passes through the patient’s body eliminating electronic noise and is, thus, presumed to deliver comparable image quality with that of previous generations of dual-source dual-energy integrating detector CT (DS-EID-CT) by significantly reduced radiation exposure or to increase image quality including spatial resolution by comparable dose exposure [10–14]. It has smaller detector elements which improve visualization of fine details (e.g., in the cancellous bone) reducing simultaneously the image noise. Other contributors to the superior image quality of DS-PCD-CT include improvements in noise reduction algorithms and iterative reconstruction techniques [15, 16]. Small detector element size may translate into improved visualization of fine detail and image noise reduction through anti-alising, particularly for high-spatial resolution images as with our protocol [17, 18].

This study, to the best of our knowledge, is the first to address the potential superiority of PCDs over energy-integrating detectors (EID) for increased spatial resolution of bone microstructure including disease-related bony defects in the clinical workup of multiple myeloma patients.

## Material and methods

### Subjects

This prospective data evaluation was approved by the institutional review board which was assigned the approval number 696/2021B01. Between October 2021 and February 2022, a total of 50 consecutive multiple myeloma patients who were referred to treatment monitoring purposes to our radiology department were enrolled. Patient consent was obtained in all cases.

Inclusion criteria were as follows: all patients underwent DS-PCD-CT using a standardized scan protocol; all patients had prior DS-EID-CT-examination performed on the same 2nd-generation DECT using also a standardized unenhanced imaging protocol with similar adjustable protocol parameters to the DS-PCD-CT. Exclusion criteria were as follows: different examinational protocols either at the DS-PCD-CT or 2nd-generation DS-EID-CT (*n* = 2) or on other scanners (*n* = 3); different examinational protocols; use of I.V. contrast agent. Eleven patients were excluded from the study.

For each patient, the levels of serum and urine M-protein were determined at the time of both CT-examinations for assessment of the degree of tumor activity. The normal values for hematologic parameters determined by the laboratory at our institution were IgG, 700–1600 mg/dL; IgA, 70–400 mg/dL; IgM, 40–230 mg/dL; serum light chains λ, 8.1–33.0 mg/L; and light chains κ, 3.6–15.9. Current myeloma-specific treatment at the time of both CT examinations was documented for each patient.

### DS-PCD-CT imaging protocol (parameters)

All examinations were performed on a 1st-generation photon-counting CT (NAEOTOM Alpha, VA40 SP1, Siemens Healthineers). Acquisition mode was ultra-high-resolution (UHR) Quantumplus, meaning an ultra-high-resolution acquisition with 2752 detector pixels (0.151 × 0.176 mm at iso-center) and 120 × 0.2 mm collimation (24 mm z-axis coverage). The UHR morphologic image information was reconstructed as T3D images from the lowest threshold. All examinations were performed without use of contrast media. Following parameters were set: 120 kVp, tube current eff. mAs 99 (current modulation by CARE Dose4D), focal spot 0.6/0.7 mm, single collimation width 0.2 mm, total collimation width 24, table speed 40.8 cm/s, table feed per rotation 20.4, spiral pitch factor 0.8, matrix size 1024 × 1024, DS-PCD-CT kernel was set at Br64 with Quantum iterative reconstruction strength Q1. Slice thickness of MPRs (sagittal and coronal) was 2 mm; single collimation width was 0.2 mm with a total collimation width of 24 mm. FOV of multiplanar reconstructions (MPR) differed according to the patient’s body size: cervical spine (125 × 175 mm), thoracic spine (300 × 325 mm), lumbar spine (125 × 145 mm), pelvis 400 × 280 mm) (Table 1).

**Table 1:** Acquisition parameters

Variables	DS-EID-CT	DS-PCD-CT
Single collimation [mm]	0.6	0.2
Total collimation [mm]	19.1	24
Table speed [cm/s]	34.8	40.8
Spiral pitch factor	0.6	0.8
Slice thickness MPR [mm]	2	2
FOV axial [mm]	500 × 500	500 × 500
FOV MPR [mm]		
Cervical spine	205 × 143	125 × 175
Thoracic spine	385 × 296	300 × 325
Lumbar spine	330 × 164	125 × 145
Pelvis	364 × 398	400 × 280
Kernel	I70f	Br64
Iterative reconstruction strength	1	Q1
Matrix size axial	512 × 512	1024 × 1024
Matrix size MPR	691 × 512	1024 × 1024

### DS-EID-CT imaging protocol (parameters)

DS-EID-CT was performed on a dual-source dual-energy scanner (SOMATOM Definition Flash, VA48A, Siemens Healthineers). The tube voltage was 100 kVp for the A tube and Sn140 kVp for the B tube. Corresponding tube current was 225 mAs (100 kVp) and 174 mAs (Sn140 kVp). Single collimation width was 0.6 mm, total collimation width was 19.1 mm, table speed 34.8 cm/s, spiral pitch factor of 0.6, kernel I70f with iterative reconstruction strength 1, and matrix 512 × 512 (axial images). Slice thickness of MPRs was 2 mm with a matrix of 691 × 512 (Table 1).

### Subjective image analysis

Image analysis was performed on a dedicated workstation (syngo.via, VA30A; Siemens Healthineers) and focused on MPRs and not axial images, as the latter were acquired with different kernels and were, thus, not optimally comparable. Therefore, MPRs were used to mitigate this at least partially, although in one direction image impression is still dominated by the kernel. A senior radiologist with 25 years of experience and a junior radiologist with 5 years of experience assessed subjective overall image quality in a blinded and independent fashion using a 5-point Likert scale: 1, very poor; 2, poor; 3, moderate; 4, good; 5, very good. Subjective image quality in terms of detectability and sharpness of bone lesions was assessed using the Likert scale depending on lesion size: smaller than 5 mm, between 5 and 10 mm, and larger than 10 mm. All cases were anonymized, randomized, and evaluated with user-adjustable windowing in separate sessions to minimize recall bias.

### Quantitative image analysis

The modulation-transfer-function (MTF) was collected for both kernels used, Br64u for DS-PCD-CT and I70 h for DS-EID-CT. The MTF is a widely used measure of resolution. The data are in line pairs per centimeter which can be translated into the corresponding object size.

### Quantification of bone microstructure (trabeculization)

**Fig. 1:**
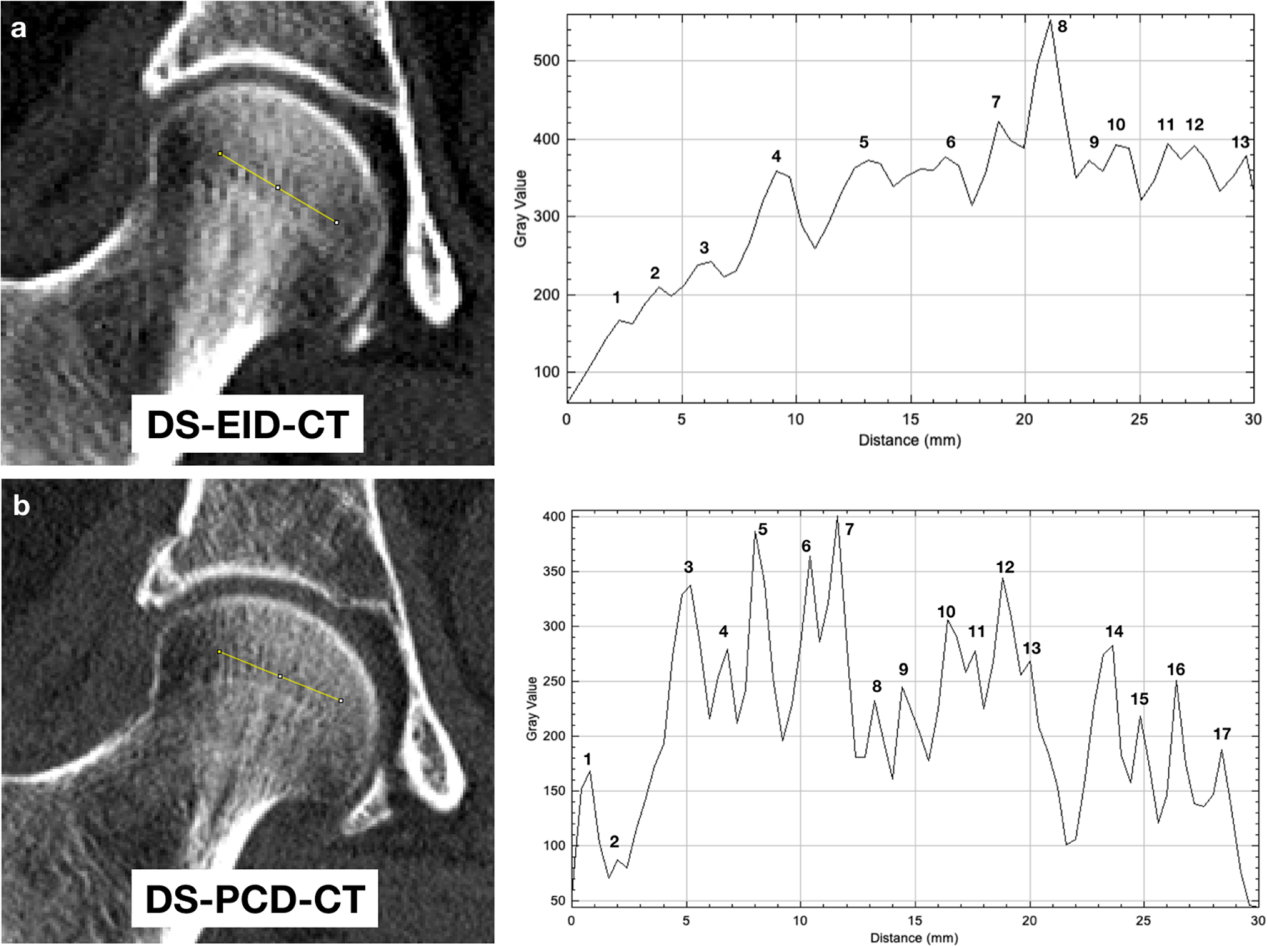
Standardized line profile in the right femoral head in coronal MPRs of the pelvis in DS-EID-CT (**a**) and DS-PCD-CT (**b**) with corresponding graphics showing plot profile of bone trabeculization. The numbered serrations correspond to the trabeculae and were obtained and evaluated for each patient. Note also the much higher blurring effect of the trabeculae on DS-EID-CT with in part indistinction of singular lines

The morphometry of the bones was analyzed using ImageJ (version 1.53 k, US National Institutes of Health, Bethesda, MD, USA). ImageJ can be used to measure distances and angles to create line profiles [19, 20]. The scan slices were imported into ImageJ software, and a standardized straight line (standardized length: 3.0 ± 0.2 cm) was segmented at a previously defined position in the spongiosa of the femoral head in all patients included in the study (Fig. 1 a + b). A plot profile was created showing a two-dimensional representation of the intensities of the pixels along the drawn line in the femoral head. The x-axis shows the length of the line and the y-axis shows the CT values. The serrations in the line profile represent the trabeculae within the spongiosa of the femoral head (Fig. 1 c + d). To measure the number of trabeculae along the standardized line, the number of serrations was counted and recorded twice by the same observer.

### Quantification of edge sharpness

**Fig. 2:**
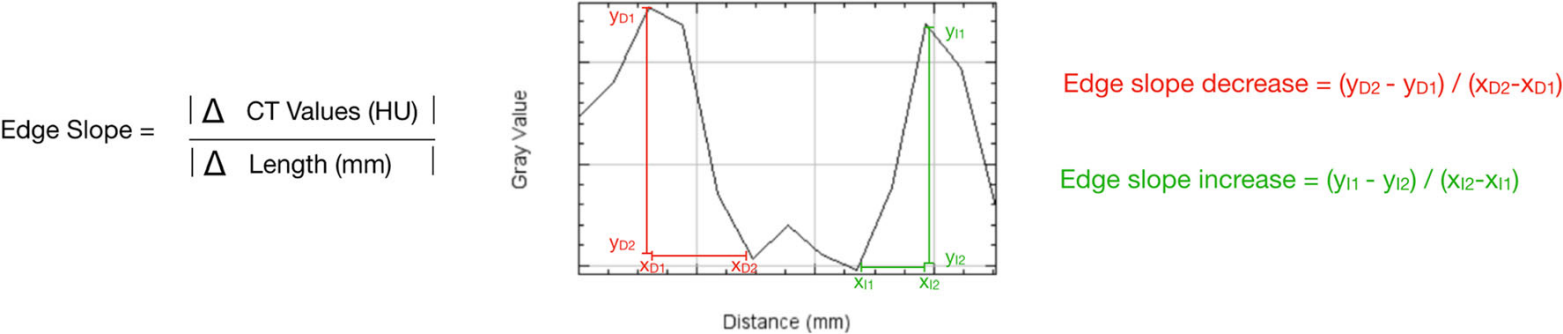
The following formula was used to quantitatively calculate the slope at the margin of the osteolytic lesions, with an example of a plot profile with the corresponding variables

Edge sharpness between the intact cancellous bone and adjacent lytic bone lesions was quantified by CT value plots along a sampling line drawn manually in sagittal or coronal MPRs at defined positions using ImageJ software. Previous studies have shown that ImageJ can successfully measure the edge sharpness of bone surfaces on CT [21]. Osteolytic lesions were identified by visual inspection with identical window-setting (bone window), and subsequently a line profile was drawn from the bone marrow of the vertebral body or pelvic bone perpendicular through the complete extent of the lytic lesion until the reentry into the regular bone surface on the opposite side of the lesion (Fig. 2 a + b). A total of 250 osteolytic lesions smaller than 5 mm and larger than 5 mm were measured in all patients who were confirmed to have osteolysis on CT. The criterion was that the lesions were the same ones on DS-EID-CT and DS-PCD-CT and that there were no size dynamics or therapeutic alterations of the bone and bone marrow at interval. For this purpose, a side-by-side analysis of submillimeter axial sizes was used for confirmation and or exclusion of new osteolysis in our study.

**Fig. 3:**
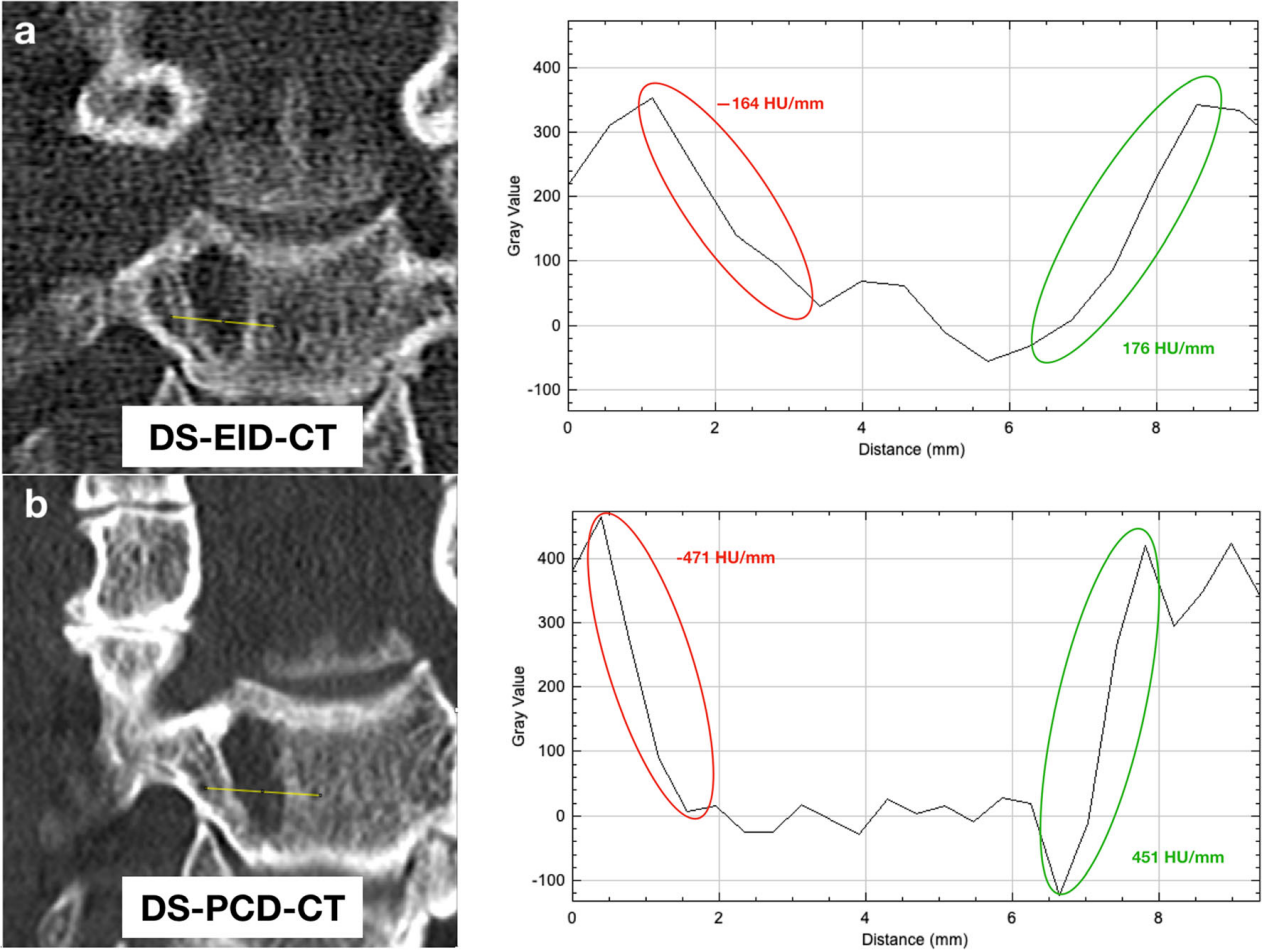
Example of a manually drawn line to quantify the edge sharpness of an osteolytic lesion in the coronary MPR of the cervical spine in DS-EID-CT (**a**) and DS-PCD-CT (**b**) with graphs corresponding to the line profile and representing the slopes of the CT values (**c** + **d**). The decrease and increase of the CT values are highlighted in red/green. Note the higher steepness of the slopes at DS-PCD-CT

The slope or gradient of the CT value plot at the entry and exit points of the osteolytic lesion was used as a measure of edge sharpness, which was defined as the difference of the CT values per millimeter [21] (Fig. 3).

### Statistics

The available data were analyzed using SPSS (SPSS Statistics, version 26, IBM Corp.) and JMP (version 14, SAS institute Inc.). Continuous variables are presented as the mean ± standard deviation, and relative frequencies are presented as *n* (%). Descriptive statistics of the data are presented as median (25th; 75th percentile or range). The Wilcoxon pairing test was used to calculate the differences in subjective image analysis. Intraclass correlation (ICC) was used to calculate the interrater agreement. ICC values ≤ 0.5 were defined as poor, those 0.51–0.75 were defined as moderate, those 0.76–0.90 were defined as good, and those > 0.90 were defined as excellent consistency. Normality of data was tested with the Shapiro-Wilk test. Data not normally distributed were analyzed with nonparametric test (Wilcoxon test). Differences of clinical status between different time points were calculated by McNemar’s chi-square test or Wilcoxon signed-rank test. A *p* value < 0.05 was defined as significant.

## Results

Between October 2021 and February 2022, 50 myeloma patients (34 male, 16 females, mean age: 67.7 ± 10.9 years) were included. Hematologic diagnosis based on M gradient was IgG (*n* = 24), IgA (*n* = 9), light chain MM (*n* = 14), and non-secretory MM (*n* = 3) (Table 2). The median time interval between clinically indicated DS-EID-CT and DS-PCD-CT was 12 months (range: 4–81 months). The radiation exposure associated with DS-EID-CT resulted in a mean dose-length product (DLP) of 1344.3 ± 204.6 mGy*cm and mean volume mean volume CT dose index (CTDIvol) of 10.1 ± 1.9 mGy. Radiation exposure related to DS-PCD-CT had a mean DLP of 1107.4 ± 247.6 mGy*cm and CTDIvol of 8.2 ± 1.8 mGy. Elevated laboratory values of serum IgG, IgA, serum or urine light-chain λ, or light-chain κ alone or in combination at date of examination were identified in 17 patients (34%) prior to DS-EID-CT and in 16 patients (32%) prior to DS-PCD-CT (*p* = 1.0). Urine M-protein was increased in 9 patients (18%) before DS-EID-CT and in 6 patients before DS-PCD-CT (*p* = 0.453). All patients with active disease experienced minimal changes in the serum (*p* = 1.0) and urine levels of M-gradient (*p* = 0.453) at interval. Most patients were in complete remission or had no disease activity at the time of DS-EID-CT (*n* = 32) and DS-PCD-CT (*n* = 33), and non-parametric analyses revealed no significant difference in disease status (complete response, partial response, and disease progression) between the two time points (*p* = 0.308). All patients with lytic bone lesions received monthly bisphosphonates. At the time of DS-EID-CT, 21 patients (42%) and during DS-PCD-CT, 19 patients (38%) received immune-based therapy. Three patients (6%) were respectively undergoing intensive cytotoxic chemotherapy at the time of DS-EID-CT and DS-PCD-CT. There was no significant difference in the distribution of the different types of treatment (*p* = 0.608) and CT imaging features (*p* = 1.0) between the two study time points. Clinical parameters on both examination days are summarized in Table 3.

**Table 2:** Patients’ demographics

Variables	*N*	%
No. of patients	50	
Age, years, mean ± SD	67.7 ± 10.9 years	
Gender		
Male	34	68
Female	16	32
Serological type		
IgG	24	48
IgA	9	18
Light-chain multiple myeloma	14	28
Non-secretory multiple myeloma	3	6

**Table 3:** Clinical parameters on both examination dates

Variables	DS-EID-CT		DS-PCD-CT	*p*-value	
	*n*	%	*n*	%	
Elevated serum IgG, IgA, serum or urine light-chain λ or light-chain κ alone or in combination	17	34	16	32	1.0
Elevated urine M-protein	9	18	6	12	0.453
Disease status					0.308
Complete response/no disease activity	32	64	33	66	
Partial response	9	18	12	24	
Progressive disease	9	18	5	10	
Treatment					0.608
Biphosphonate	41	82	42	84	
Immune-based therapy	21	42	19	38	
Intensive cytotoxic chemotherapy	3	6	3	6	
CT imaging features					1.0
Infiltration of marrow space	7	14	5	10	
Normal bone marrow	9	18	8	16	
Lytic lesions	41	82	42	84	
Number of lesions: 1–3	6	12	7	14	
More than 3 lytic lesions and/or pathologic fracture	35	70	35	70	

### Subjective image analysis

Likert score ratings for overall image quality were significantly superior for both readers for DS-PCD-CT compared with measurements for DS-EID-CT (all *p* < 0.0001).

Likert scores for detectability and sharpness of bone lesion edges were significantly higher with DS-PCD-CT compared to DS-EID-CT (all *p* < 0.0001). Detectability and sharpness of lesions were independent of lesion size (*p* = 0.1–1.1) (Table 4).

**Table 4:** Overview of data from qualitative image analysis

Parameter	Pelvic bone			Cervical spine			Thoracic spine			Lumbar spine		
	DS-EID-CT	DS-PCD-CT	*p*-value	DS-EID-CT	DS-PCD-CT	*p*-value	DS-EID-CT	DS-PCD-CT	*p*-value	DS-EID-CT	DS-PCD-CT	*p*-value
Overall image quality												
Reader 1	4 (3–4)	5 (5–5)	< 0.0001	3 (2–3)	4 (4–5)	< 0.0001	4 (3–4)	5 (4–5)	< 0.0001	4 (3–4)	5 (4–5)	< 0.0001
Reader 2	4 (3–4)	5 (5–5)	< 0.0001	3 (2–3)	4 (4–5)	< 0.0001	4 (3–4)	5 (4–5)	< 0.0001	4 (3–4)	5 (4–5)	< 0.0001
Detectability of bone lesions												
Reader 1												
Lesion size												
< 5 mm	4 (4–4)	5 (5–5)	< 0.0001	3 (3–4)	4 (4–5)	< 0.0001	4 (3–4)	4 (4–5)	< 0.0001	4 (3–4)	5 (4–5)	< 0.0001
5–10 mm	4 (3–4)	5 (5–5)	< 0.0001	3 (3–4)	4 (4–5)	< 0.0001	4 (3–4)	5 (4–5)	< 0.0001	4 (3–4)	5 (4–5)	< 0.0001
> 10 mm	4 (3–4)	5 (5–5)	< 0.0001	3 (3–3)	4 (4–5)	< 0.0001	3 (3–4)	5 (5–5)	< 0.0001	4 (3–4)	5 (5–5)	< 0.0001
Reader 2												
Lesion size												
< 5 mm	4 (4–4)	5 (5–5)	< 0.0001	3 (3–3)	4 (4–5)	< 0.0001	3 (3–4)	5 (4–5)	< 0.0001	4 (3–4)	5 (4–5)	< 0.0001
5–10 mm	4 (3–4)	5 (5–5)	< 0.0001	3 (3–3)	4 (4–5)	< 0.0001	4 (3–4)	5 (4–5)	< 0.0001	4 (3–4)	5 (4–5)	< 0.0001
> 10 mm	4 (3–4)	5 (5–5)	< 0.0001	3 (2–3)	4 (4–5)	< 0.0001	4 (3–4)	5 (5–5)	< 0.0001	4 (4–4)	5 (5–5)	< 0.0001
Delineation (sharpness) of bone lesions												
Reader 1												
Lesion size												
< 5 mm	4 (3–4)	5 (5–5)	< 0.0001	3 (2–3)	4 (4–5)	< 0.0001	3 (3–3)	5 (5–5)	< 0.0001	3 (3–4)	5 (5–5)	< 0.0001
5–10 mm	4 (3–4)	5 (4–5)	< 0.0001	3 (3–3)	4 (4–5)	< 0.0001	3 (3–4)	5 (5–5)	< 0.0001	3 (3–4)	5 (5–5)	< 0.0001
> 10 mm	4 (3–4)	5 (5–5)	< 0.0001	3 (2–3)	5 (4–5)	< 0.0001	3 (3–4)	5 (5–5)	< 0.0001	3 (3–4)	5 (5–5)	< 0.0001
Reader 2												
Lesion size												
< 5 mm	3 (3–4)	5 (5–5)	< 0.0001	3 (2–3)	4 (4–5)	< 0.0001	3 (3–4)	5 (5–5)	< 0.0001	3 (3–4)	5 (5–5)	< 0.0001
5–10 mm	3 (3–4)	5 (4–5)	< 0.0001	3 (2–3)	4 (4–5)	< 0.0001	3 (3–4)	5 (5–5)	< 0.0001	3 (3–4)	5 (5–5)	< 0.0001
> 10 mm	3 (3–4)	5 (4–5)	< 0.0001	3 (2–3)	5 (4–5)	< 0.0001	3 (3–4)	5 (5–5)	< 0.0001	3 (3–4)	5 (5–5)	< 0.0001

Data are presented as median qualitative image analysis score; data in parentheses are interquartile ranges. *DS-EID-CT*, dual-source dual-energy integrating detector CT; *DS-PCD-CT*, dual-source photon-counting detector CT

Interreader agreement showed excellent consistency between readers 1 and 2 for the metrics’ overall image quality, detectability of lytic lesions, and sharpness of lytic lesions (*α* values ranged from 94.2 to 98.8).

### Quantitative image analysis

MTF values for the reconstruction kernels used as a direct technical measurement of spatial resolution are outlined in Table 5. Based on the 2% MTF, this results in the minimum detectable object size of 0.36 mm for the Br64u kernel and 0.41 mm for the I70 h kernel.

**Table 5:** Modulation-transfer function values for the employed reconstruction kernels

MTF in Ip/cm	Br64u	I70h
50%	10.1	9.2
10%	12.7	11.3
2%	14.0	12.3

*MTF*, modulation-transfer-function; *Ip/cm*, line pairs per centimeter

### Quantification of trabeculization

**Fig. 4:**
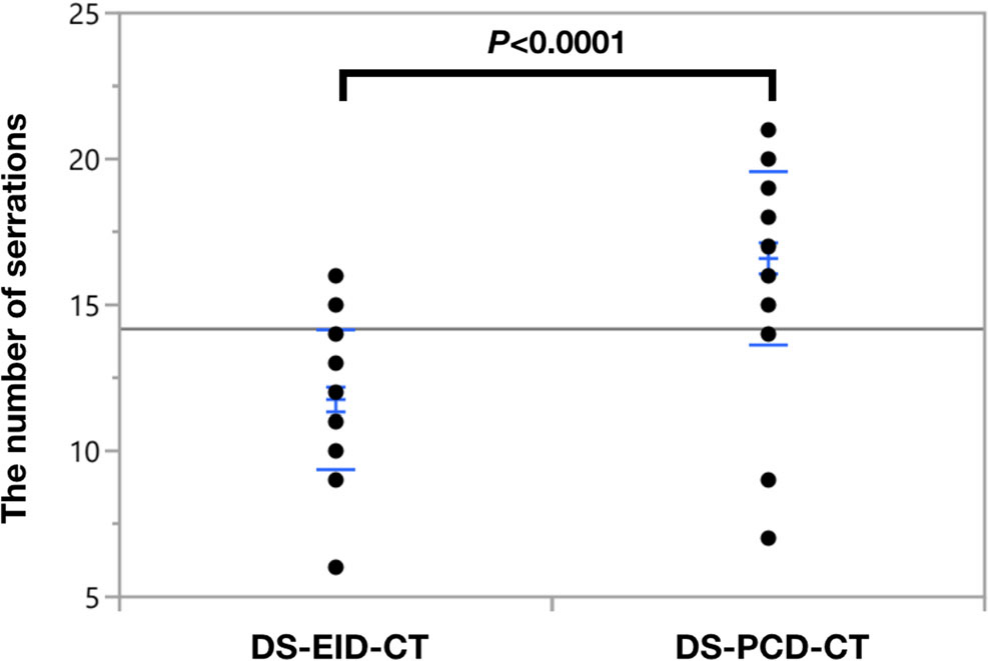
The number of serrations on plot profiles of DS-EID-CT and DS-PCD-CT

Quantification of trabeculization of bone in the femoral head revealed that serrations were significantly more frequent in DS-PCD-CT MPRs than in DS-EID-CT MPRs (16.6 ± 2.9 vs. 11.8 ± 2.4, *p* < 0.0001) (Fig. 4). Depending on the length of the line profile, 5.5 ± 0.9 serrations per cm were detected in DS-PCD-CT and 3.9 ± 0.7 serrations per cm in DS-EID-CT (*p* < 0.0001).

### Quantification of edge sharpness

**Fig. 5:**
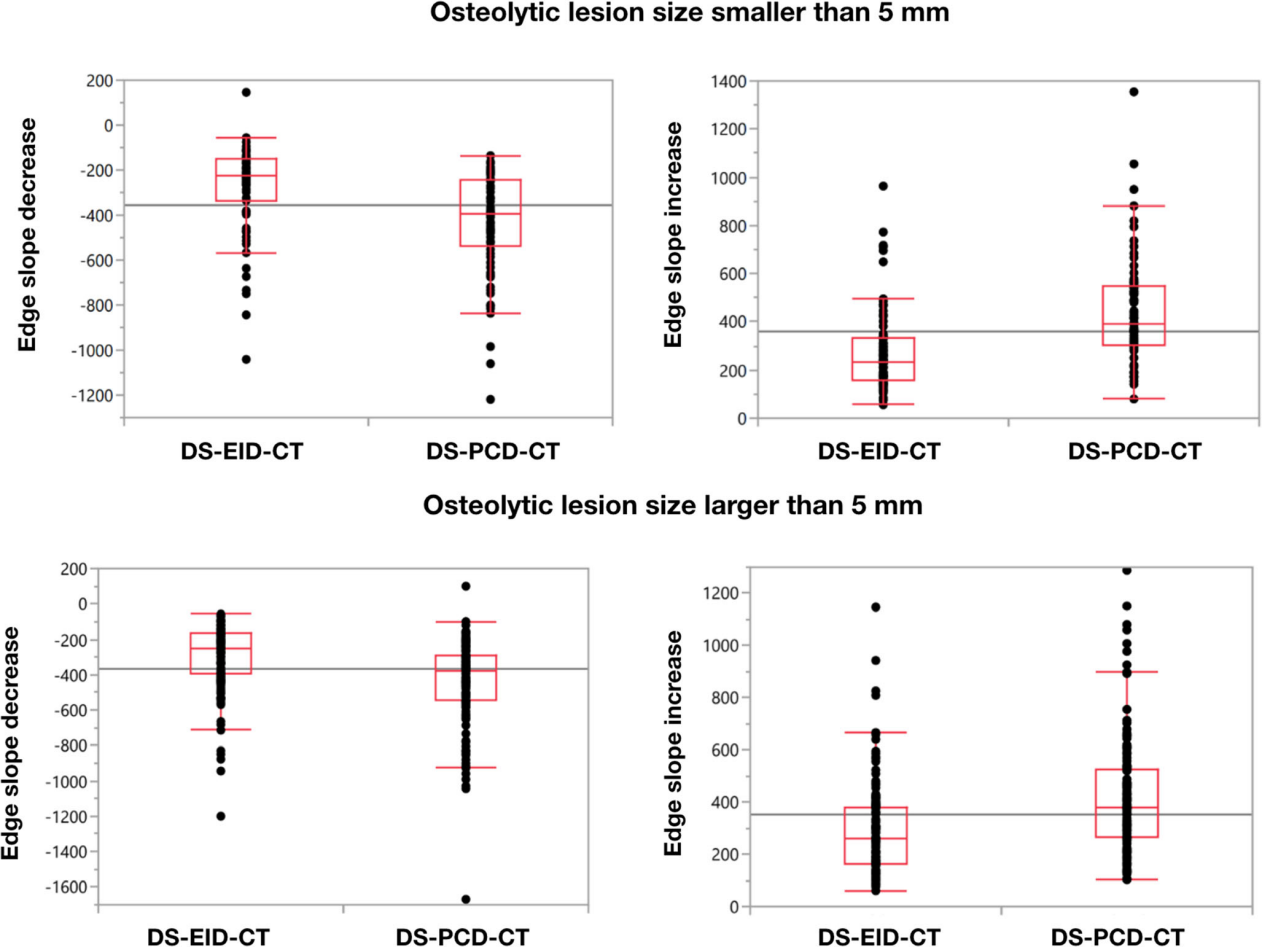
Boxplots show median and quantiles of edge slope decrease and increase for osteolytic lesions smaller and larger than 5 mm

Quantitative analyses of edge sharpness of osteolysis showed significant differences between DS-EID-CT and DS-PCD-CT with consistently steeper edges for DS-PCD-CT (all *p*'s < 0.001). For lesions smaller than 5 mm, DS-PCD-CT measured steeper values compared to DS-EID-CT both for decrease (median [interquartile range]: −393 [−537.5, −243.0] vs. −225 [−335.5, −149.0]) and increase (388 [300.5–548.5] vs. 234 [115.8–334.0]) in slope. For osteolytic lesions larger than 5 mm, DS-PCD-CT also showed steeper values in comparison with DS-EID-CT for decrease (−378 [−545.5, −288.0] vs. −253 [−391, −163) and increase (379 [−266.0, −526.0] vs. 258 [163.0–377.0]) in slope (Fig. 5).

## Discussion

In this study, an ultra-high-resolution scan mode on a DS-PCD-CT scanner with a detector pixel size of 0.2 mm at the isocenter was compared with a DS-EID-CT with a detector pixel size of 0.2 × 0.2 mm^2^ for whole-body skeletal imaging in patients with multiple myeloma focusing on the trabecular microstructure of the cancellous bone and on the disease-related lytic bone lesions. The scan parameters were set as far as possible at similar levels for both scanners in order to achieve similar radiation doses which finally did not differ significantly from each other although they proved slightly lower (10–15%) for the DS-PCD-CT. As on DS-EID-CT only the sagittal and coronal MPRs were reconstructed using a sharp kernel, we decided to focus our image analysis for both scanners on these reformates due to the better comparability of these image kernels. Most other adjustable parameters were also similar.

The results of our study show significant qualitative and quantitative image quality improvement on the DS-PCD-CT compared to the DS-EID-CT. Hence, the delineation of bony trabeculae as well as the sharpness of their edges and the transitions between the cancellous bone and focal lytic bone lesions was more easily and reliably accomplished on DS-PCD-CT. Notably, this trend stayed unimpaired even in bone lesions smaller than 5 mm. Despite the use of a sharp kernel and submillimeter single slice collimation, depiction of densely packed trabeculae in the femoral heads exhibited a blurred, unsharp appearance on the DS-EID-CT. Knowingly, the trabecular density varies significantly between subjects being dependent on many factors like age, gender, bone metabolism, medication, physical activity, etc. Therefore, only an intra-individual comparison is allowed in such cases which we have accomplished in this study showing an interreader agreement of between 94.2 and 98.8%.

For more objective data analysis, we additionally compared the delineation (sharpness) of the contours of lytic bone lesions by the maximal slope within CT value plots and found a significantly steeper slope with DS-PCD-CT as with DS-EID-CT. Here again, quantitation of the number of serrations (trabecular structures) was significantly higher for DS-PCD-CT compared to DS-EID-CT and these results were proven once more independent on the lesion size. This latter aspect may lead in the clinical routine while comparing CT-images from different scanners to the false visual impression of detecting more bone lesions on the DS-PCD-CT because they become more conspicuous. A side-by-side analysis of submillimeter axial sizes was used for confirmation and or exclusion of new osteolysis in our study.

Improvement of spatial resolution has been always in the focus of radiological imaging. Different approaches have been proposed and tested in the clinical routine; however, the main limitations with the conventional DS-EID-CT are the radiation dose and the size of the detector elements. The photon-counting detectors have opened new possibilities for improved spatial resolution without loss of dose efficiency. Conducting a study on a DS-PCD-CT using two high-resolution scan modes (sharp and UHR), Leng et al reported a 69% and 87% improvement in in-plane resolution over an EID-scanner for a detector pixel size of 0.25 mm at the isocenter [12]. The use of filters consisting of many septa decreases the size of detector elements with EIDs, but at the same time it increases dose as the blocked photons have already contributed to patient dose, but not to the reconstructed image. On the contrary, no septa are present in DS-PCD-CT and therefore reduced efficiency is not expected. The superiority of the DS-PCD-CT scanner for decreasing image noise could be already demonstrated in a cadaveric study by Zhou et al, focusing on temporal bone imaging [22]. These authors indicated the potential of a 64% reduction in dose using a DS-PCD-CT UHR mode for clinical bone imaging. Besides first attempts to improve spatial resolution with aid of PCD-technology for smaller structures (small FOV), Rajendran et al reported their initial experience with a DS-PCD-CT using a full FOV in UHR mode (0.2 mm resolution) [23]. Their study reported a 37% lower radiation dose and 46% lower image noise compared with EID-CT. The authors thus demonstrated that larger body parts can be imaged in the UHR mode. Similarly, but in a phantom study, Yu et al investigated and confirmed the clinical feasibility of a whole-body DS-PCD-CT-scanner [24]. Using the same quantification software, Bette et al, in an animal study, demonstrated the subjective and objective image quality superiority of DS-PCD-CT over DS-EID-CT for delineation of tiniest bone details [13]. Thus, PCD improves image quality and also spatial resolution because of the more efficient conversion of x-rays into light and by minimizing electron noise [25]. Also improvements with the iterative reconstruction techniques contribute to the observed improvements in image quality [15]. In line with these previous reports, our study shows that a significant improvement in spatial resolution is possible with the new DS-PCD-CT by comparable or lower radiation dose as with DS-EID-CT even for whole-body studies with full FOV.

Our study has some limitations. First, the number of patients was too small in order to allow for a more sophisticated subgroup analysis of patients with different degrees of disease activity affecting the contrast between the infiltrated bone marrow in the background and the focal osteolysis. Second, we did not have additional information on the underlying bone density of our patients which would be expected to also impact the contrast between bone marrow and trabeculae of the cancellous bone as well as their thicknesses. Third, slight differences with respect to the used kernels as well as the matrix could have had a certain impact on image comparability, but at least the former is no more available for the DS-PCD-CT. We used MPRs to mitigate the differences in the kernels, but in either the x- or y-direction, the image impression is still dominated by the kernel.

To the best of our knowledge, this study is the first to confirm the superiority of DS-PCD-CT over DS-EID-CT for assessment of bone microstructure in humans, in particular in the clinical setting of myeloma bone disease.

In conclusion, DS-PCD-CT significantly improves spatial resolution of bony microstructure and disease-related (lytic bone lesions) compared to 2nd-generation DS-EID-CT.

## Abbreviations

CT: Computed tomography
CTDI: Computed Tomography Dose Index
DLP: Dose length product
DS-EID-CT: Dual-source dual-energy integrating detector CT
DS-PCD-CT: Dual-source photon-counting detector CT
EID: Energy-integrating detectors
EID-CT: Energy-integrating detector CT
FOV: Field of view
ICC: Intraclass correlation
IgA: Immunoglobulin A
IgG: Immunoglobulin G
MPR: Multiplanar reconstruction
MRI: Magnetic resonance imaging
MTF: Modulation transfer function
PCD: Photon-counting detector
UHR: Ultra-high-resolution

## Declarations

### Guarantor

The scientific guarantor of this publication is Dr. Moritz Winkelmann.

### Statistics and biometry

Dr. Moritz Winkelmann has significant statistical expertise.

### Methodology

• prospective

• diagnostic or prognostic study

• performed at one institution
